# Reported dietary intake in early pregnant compared to non-pregnant women – a cross-sectional study

**DOI:** 10.1186/s12884-014-0373-3

**Published:** 2014-11-01

**Authors:** Anette Lundqvist, Ingegerd Johansson, AnnaLena Wennberg, Johan Hultdin, Ulf Högberg, Katarina Hamberg, Herbert Sandström

**Affiliations:** Department of Public Health and Clinical Medicine, Family Medicine, Umeå University, Umeå, Sweden; Department of Odontology, Cariology, Umeå University, Umeå, Sweden; Department of Medical Biosciences, Umeå University, Umeå, Sweden; Department of Women’s and Children’s Health, Uppsala University, Uppsala, Sweden

**Keywords:** Pregnancy, Diet, Nutrition, Cross-sectional

## Abstract

**Background:**

A woman’s nutritional status before conception and during pregnancy is important for maternal health and the health of the foetus. The aim of the study was to compare diet intake in early pregnant women with non-pregnant women.

**Methods:**

Between September 2006 and March 2009, 226 women in early pregnancy were consecutively recruited at five antenatal clinics in Northern Sweden. Referent women (n = 211) were randomly selected from a current health screening project running in the same region (the Västerbotten Intervention Program; VIP). We collected diet data with a self-reported validated food frequency questionnaire with 66 food items/food aggregates, and information on portion size, alcohol consumption, and supplement intake. Data were analysed using descriptive, comparative statistics and multivariate partial least square modelling.

**Results:**

Intake of folate and vitamin D from foods was generally low for both groups. Intake of folate and vitamin D supplements was generally high in the pregnant group and led to significantly higher total estimated intake of vitamin D and folate in the pregnant group. Iron intake from foods tended to be lower in pregnant women although iron supplement intake evened out the difference with respect to iron intake from foods only. Energy intake was slightly lower in pregnant women but not significant, a reflection of that they reported consuming significantly less of potatoes/rice/pasta, meat/fish, and vegetables (grams/day) than the women in the referent group.

**Conclusions:**

In the present study, women in early pregnancy reported less intake of vegetables, potatoes, meat, and alcohol than non-pregnant women. As they also had a low intake (below the Nordic Nutritional Recommendations) of folate, vitamin D, and iron from foods, some of these women and their unborn children are possibly at risk for adverse effects on the pregnancy and birth outcome.

## Background

A woman’s nutritional status before conception and during pregnancy is important for maternal health and the health of the foetus; in addition, poor nutritional status can lead to the child developing disease later in life [1,2]. The Nordic Nutritional Recommendations (NNR) recommends energy and nutrient intake for the general population, including pregnant and lactating women [3]. The NNR focuses on dietary patterns and food quality for good health and prevention of diet-related chronic diseases. In the first trimester of pregnancy, the NNR recommends an addition of 200 kcal/day, although other sources recommend an addition of 300–400 kcal/day [4]. Equally important as the increased need for energy during early pregnancy is the elevated need for various micronutrients [5,6]. The NNR recommends that pregnant women increase their folate intake by 65%, selenium by 40%, iron, zinc, and vitamin D by 25 - 30%, and calcium, phosphorus, and most other minerals and vitamins by 20% [3]. In a Swedish survey on diet intake conducted in 2010 to 2011, childbearing-aged women had lower than recommended intake of fruit, berries, vegetables, and whole grain products, but a higher than recommended intake of fatty foods and sugar and lower than recommended intake of folate, vitamin D, and iron [7]. This picture indicates a potential risk if the woman becomes pregnant. Similar studies focusing on early pregnant Caucasian women in Great Britain have shown inadequate intakes of vitamin C, folate, calcium, and iron [8–10]. Women with unplanned pregnancies, young mothers, and those with low socioeconomic status were particularly prone to low intake levels [9]. These findings are especially concerning as low folate intake can increase the risk of neural tube defects. Low folate levels are also suggested to be associated with other birth defects, such as cleft lip and palate, heart defects, and autism [11,12], and low levels of vitamin D are associated with various adverse pregnancy and birth outcomes [13,14]. Thus, the low intake of folate, vitamin D, and iron found in Swedish women who are of childbearing age is of special concern, as women do not seem to improve the quality of their overall diet during pregnancy [7,15]. In addition, maternal obesity has increased in recent decades in Western countries, a condition that increases the risk of several complications for both the mother and infant [16,17]. Thus, a woman’s lifestyle in the early phase of pregnancy, including her dietary habits, should be part of the antenatal care counselling by health professionals. Knowledge on the dietary habits of women in early pregnancy should underpin such counselling in the first phases of antenatal care to prevent adverse effects later in pregnancy.

The aim of the present cross-sectional population-based study was to compare dietary patterns of early pregnant women with non-pregnant women in the county of Västerbotten, Northern Sweden.

## Methods

### Pregnant group

The present study is a cross-sectional survey of 226 women in early pregnancy recruited from five antenatal clinics in primary care in Umeå, Sweden. Participants, who were consecutively recruited between September 2006 and March 2009, were part of a longitudinal study (PregNut) where dietary intake, height, and weight were measured and blood sampled during pregnancy and postpartum. One hundred and forty-three of the women were nullipara, 83 multipara, 222 had a singleton pregnancy, and four had a twin pregnancy. All these women but ten were born in Sweden, 223 were married or cohabiting, and three were single. All women attending the antenatal clinics were invited by midwives, and women who expressed an interest in participating were given verbal and written information. Signed consent was obtained during the first visit to the antenatal welfare program. Three exclusion criteria were used: major medical conditions, unable to attend the ordinary antenatal welfare program, and insufficient competence in the Swedish language.

### Dietary measures

The pregnant women were asked to answer a 66-item food frequency questionnaire (FFQ). The FFQ is a shortened version of the original Northern Sweden FFQ, but the questions used were the same as the original version. Both the original and shortened versions were designed to be semi-quantitative and optically readable for data input. The Northern Sweden FFQ is used by the Västerbotten Intervention Programme (VIP) [18], the European Prospective Investigation into Cancer and Nutrition (EPIC) [19], and the Northern Sweden WHO Multinational Monitoring of Trends and Determinants in Cardiovascular Disease (MONICA) [20]. The original Northern Sweden FFQ has been validated against ten repeated 24 h dietary recalls and selected plasma or erythrocyte biomarkers with regard to intake of food, energy, and macronutrients, vitamins, minerals, and fatty acids [18,21,22]. The correlation coefficients for the two recording methods were typically between 0.45 and 0.61, and the median correlation coefficient for all nutrients was 0.50. Consumption frequencies were reported on a nine-level scale, from 0 (never) to 8 (4 times/day or more). The shortened version included eight questions on the frequency of consumption of various types of fats used for spreading on bread or cooking, nine on milk and other dairy products, seven on bread and cereals, six on fruit, greens and root vegetables, and six on soft drinks and sugar-containing snacks. Five questions on spirits, wine and beer consumption were included in a list of beverages. Twenty of the remaining 25 questions recorded, intake of potato, rice, pasta, meat and fish, and five were on varied items, such as salty snacks, coffee, tea and water. The participants indicated their average portion of (*i*) potato/pasta/rice, (*ii*) vegetables, and (*iii*) meat/fish using four colour photographs illustrating four plates with increasing portion sizes of potatoes, vegetables, and meat. For other food items, either gender and age portion sizes or standard portion sizes were used as described previously [17]. The reported consumption frequencies were converted to number of intakes per day. The content of energy and nutrients was calculated by multiplying daily intake frequency by the portion content according to the latest available update for the specific nutrient in the database provided by the National Food Administration (Uppsala, Sweden) [23]. In general the shorter version yields lower total energy intake reports but rank subjects in the same order [21].

Five pregnant women did not answer the FFQ and 12 more had either (*i*) left ≥10% food questions unanswered or (*ii*) lacked at least one portion size indication or (*iii*) had an estimated food intake level (FIL, reported energy intake/basal metabolic rate) corresponding to the lowest 5% and highest 2.5% as described earlier [24]. These women were excluded, leaving 209 pregnant women for diet intake evaluation. For these women, mean (99% CI) number of days of pregnancy at time of examination was 85 days (82–87 days).

### Other measures

The participants also completed a questionnaire, which in addition to diet intake, covered socioeconomic and psychosocial conditions, marital status, level of education, self-rated health, personal health history, family history, and quality of life [25]. The questionnaire also covered social network and support, working conditions, physical activity, alcohol consumption, tobacco use, and dietary supplement use. For supplements, intake of multimineral, multivitamin, and iron supplement was reported as “Yes” or “No” for intake during the previous two weeks. In addition, body weight (light clothing) and height (no shoes) were measured.

### Referent group

A group of referent women was nested in the current Västerbotten Intervention Program (VIP) [18]. VIP invites all 40-, 50-, and 60-year-old inhabitants in Västerbotten County to a health screening. In some communities, 30-year olds are also invited. The Northern Sweden Diet Database (NSDD) compiles diet data for the VIP, producing approximately 140 000 observations [26]. All 30-year-old women from the larger Umeå area, who had participated in VIP during the same recruitment period as the pregnant women in this study, were included as referents (n = 108). Of these, five women who did not fulfil the FFQ quality criteria described above were excluded. An equally sized group of 40-year-old women (n = 103) in the NSDD with FFQs fulfilling the quality criteria – being from the Umeå area and attending VIP in the same period as the pregnant women in this study – was randomly selected as referents for 40-year-old pregnant women. The referent women also had their height and weight measured during their VIP and completed the same questionnaire as the pregnant women. Thus, information for the referents and pregnant women was obtained using an identical questionnaire and virtually identical routines. The only difference was that the referent women completed the FFQ from the perspective of intake the previous year but not just the previous two weeks.

### Data handling and statistical analysis

As a basis for data analysis in the present project, the distribution of reported energy and nutrient intake was evaluated among all 30- and 40-year-old women in NSDD with a diet recording fulfilling FFQ quality criteria and with a screening date within the same period as recruitment of the pregnant women in this study (n = 26 394). The distribution was found to be acceptably normal for all diet variables, except alcohol. Thus, for descriptions, means with 95% confidence interval (CI) are presented for dietary variables, except alcohol intake where median with max-min values are presented. Intake of total fats, carbohydrates, proteins, and alcohol are presented as the proportion energy they provide in per cent of the total reported energy intake (E%). To compensate for the systematic underreporting by the shortened FFQ, reported intakes were extrapolated to an energy level corresponding to the 25% reduction of the original FFQ.

Differences between groups were tested with Student’s *t*-test for normally distributed variables after appropriate adjustments. Mann–Whitney *U* test was used to test the difference in alcohol intake. In addition to testing for differences in estimated amounts eaten per day, differences in residuals from regressions of the respective nutrient on energy intake was assessed for vitamins and minerals as described by Willet [27]. All residuals were normally distributed. The use of residuals was done to circumvent potential errors from underreporting or over-reporting. In the present population, underreporting has been found common due to the instrument, high BMI, low education, and smoking [28]. Accordingly, for ten-year age groups, means of nutrient intakes were calculated by standardizing for BMI groups, education level, and smoking. When comparing all pregnant and referent women, age group was also included as a covariate.

Participants were classified as normal weight (BMI <25), overweight (BMI ≥25 - <30), or obese (BMI ≥30). No pregnant or referent women had a BMI <18. Use of tobacco (smoking or Swedish snus (snuff)) and alcohol was dichotomized into present-use or no-use. Education was dichotomized into having a university education or not. Physical activity was dichotomized as having a low physical activity at work, leisure-time, both or neither. Differences in the distributions of these variables and proportions of obese, overweight, and normal-weight subjects among pregnant versus referent women were tested with a Chi-square test, unless the number in a cell was five or lower, in which case Fisher’s exact test was used.

Use of a supplement was dichotomized into taking a supplement the previous two weeks or not. Vitamin and mineral supplementation was estimated by using the most frequent content in over-the-counter preparations aimed for women. Thus, the following additions were made: calcium, 200 mg; iron, 18 mg; vitamin D, 7.5 μg; vitamin B12, 2.5 μg; and folate, 200 μg. The latter was adjusted for increased bioavailability from folic acid by the factor 1.7 [29]. P-values <0.01 were considered statistically significant. IBM SPSS Statistics version 20 was used for these data analyses.

Multivariate partial least square modelling (PLS) was performed to search for clustering among the women and to identify variables associated with being early pregnant or a referent woman. The software SIMCA P+, version 12.0 (Umetrics AB, Umeå, Sweden) was used. The independent (X) block included the 66 foods/aggregates in the FFQ, estimated nutrients, supplement use, and tobacco use. Variables were autoscaled to unit variance, and cross-validated prediction of Y was calculated. Clustering of participants was displayed in a score loading plot with the two strongest components (t[1] and t[2]) on the x- and y-axis [30,31]. For the theoretical concept behind PLS modelling we refer to the review paper by Haenlein and Kaplan [32].

### Ethical approval

The study was approved by the Regional Ethical Review Board at Umeå University Sweden (Dnr 04-171 M). The clinics involved are all part of Västerbotten County Council and under the evaluation by Regional Ethical Review Board at Umeå University. Thus, the given ethical approval from the Regional Ethical Review Board at Umeå University Sweden includes all health centers.

## Results

Characteristic of the early pregnant women and comparisons with the referent women are presented in Table 1. Compared to the referent group, a larger proportion of the pregnant women had university education, a lower proportion smoked or used snuff, and a higher proportion abstained from alcohol, and consumed amounts of alcohol per day was significantly lower. All other evaluated aspects were similar in the pregnant and referent group. Comparisons between ten-year age groups revealed no major difference for the pregnant women but did so for some variables in the referent group (Table 1).

**Table 1:** **Characteristics in early pregnant women and a population based referent group**

	**Pregnant women**			**Referent women**			**Pregnant vs referent women**
	**<35 years**	**≥35 years**		**<35 years**	**≥35 years**		
	**(n = 176)**	**(n = 33)**	***p*** **-value**	**(n = 103)**	**(n = 103)**	***p*** **-value**	***p*** **-value**
Age (mean (95% CI))^1^	29.2 (28.7 –29.7)	37.0 (36.4-37.6)	-	30.1 (30.0-30.2)	40.0 (40.0 – 40.0)	-	<0.001^4^
Married or cohabitant (%)^2^	94.5	100	0.359	90.3	75.5	0.001	0.302
Education (% with university)^2^	59.5	77.4	0.059	35.9	60.8	<0.001	<0.001
BMI (mean (95% CI))^1^	24.1 (23.3-24.8)	25.0 (23.3-26.8)	0.330	25.9 (24.8-26.9)	24.5 (23.4-25.6)	0.085	0.179
Normal weight BMI < 24.9 (%)^2^	71.4	61.3	0.354	54.4	68.9	0.099	0.096
Overweight BMI ≥25.0-29.9 (%)	20.5	32.3		29.1	19.4		
Obese BMI ≥ 30.0 (%)	8.1	6.5		16.5	11.7		
Smoking (% smoker)^2^	0.0	3.2	0.160	6.8	5.8	0.774	0.017
Snuff use (% user)^2^	1.2	0.0	1.000	17.5	7.8	0.036	<0.001
Alcohol g/day (median (min – max))^3^	0.11 (0–15.0)	1.8 (0–13.7)	0.028	1.8 (0–21.2)	2.5 (0–19.4)	<0.001	<0.001
Alcohol No (%)^2^	38.6	21.2	0.041	12.6	4.9	0.040	<0.001
Low physical activity at work (%)^2^	27.3	45.5	0.037	25.2	34.0	0.170	0.330
Low physical activity at leisure activity (%)^2^	57.1	60.0	0.771	56.3	38.9	0.012	0.237
Low physical activity at work and leisure time (%)^2^	15.9	27.3	0.117	21.4	15.5	0.281	0.868

^1^Differences between means were tested with two-sided Student’s *t*-test. Means for BMI in age groups were adjusted for education level using a generalized linear model. For comparisons between all pregnant and referent women mean values were adjusted for education and age group using a generalized linear model.

^2^Differences in sampling distribution were tested with Pearson’s Chi-square test or Fischer’s exact test if five or fewer observations in a cell; For BMI groups (normal weight, overweight, obesity) differences in sample distribution were tested among all tree levels.

^3^Data, which are based on FFQ information, were not normally distributed. Differences between age groups were tested with Mann–Whitney *U* test.

^4^The difference in mean age between pregnant and referent women was statistically significant in both age strata (p < 0.01).

Intakes of energy and energy-providing components (carbohydrates, fats, proteins, and alcohol) and some selected nutrients for the early pregnant and referent women are shown in Table 2. Overall, the estimated intake of macro- and micronutrients was similar for pregnant women under 35 and those aged 35 years and over (Table 2). When both age groups were evaluated together, early pregnant women reported slightly lower, although not statistically significant (p > 0.01), intake of energy than referent women. After adjustment for the anticipated underreporting due to the 25% reduction of the FFQ compared to the original FFQ, the estimated mean energy intake was 2033 and 1911 kcal/day compared to 2144 and 2102 kcal/day in the <35-year-old and ≥35-year-old pregnant and referent women, respectively. The slightly lower energy intake in pregnant women reflected that they reported a significant less (grams/day) consumption of potato/rice/pasta, meat/fish, and vegetables than the women in the referent group (Table 2).

**Table 2:** **Reported and estimated daily energy, nutrient and food group intake**

	**Pregnant women**			**Referent women**			**Pregnant vs referent women**	
**Daily intake**	**<35 years**	**≥35 years**		**<35 years**	**≥35 years**		***p*** **-value**	
							**Based on means**	**Based on residuals** ^**6**^
	**(n = 176)**	**(n = 33)**	***p*** **-value**	**(n = 103)**	**(n = 103)**	***p*** **-value**		
**Nutrients**								
Reported total energy (kcal)^1^	1627 (1550–1703)	1529 (1345–1712)	0.334	1715 (1626–1804)	1682 (1592–1772)	0.612	0.013	-
Carbohydrate (E%)^1^	45.7 (44.8-46.7)	46.9 (44.6-49.1)	0.353	45.2 (44.0-46.5)	48.0 (46.8-49.3)	0.705	0.649	-
Fat (E%)^1^	38.9 (37.9.2-39.9)	36.1 (33.7-38.4)	0.028	38.8 (37.5-40.1)	34.2 (32.9-35.5)	0.215	0.183	-
Protein (E%)^1^	14.7 (14.4-15.0)	15.7 (15.0-16.5)	0.017	15.0 (14.5-15.5)	16.1 (15.6-16.6)	0.264	0.259	-
Alcohol (E%)^1^	0.5 (0.4-0.7)	1.2 (0.8-1.6)	0.003	0.9 (0.6-1.1)	1.4 (1.1-1.7)	0.022	0.019	-
Estimated total energy (kcal)^2^	2033 (1938–2130)	1911 (1681–2141)		2144 (2032-2256	2102 (1990–2215)		-	
Whole grain (g)^2^	57.6 (54.2-61.1)	76.6 (68.4-84.8)	<0.001	61.8 (56.6-67.0)	75.8 (70.5-81.0)	<0.001	0.784	0.960
Sucrose (g)^2^	32.4 (30.3-34.5)	27.8 (22.8-32.9)	0.105	33.4 (30.4-36.3)	30.7 (27.7-33.7)	0.232	0.810	0.985
Total fat (g)^2^	86.5 (83.6-89.5)	79.1 (72.0-86.2)	0.059	91.3 (87.3-95.2)	78.4 (74.4-82.4)	<0.001	0.561	0.173
Saturated fatty acids (g)^2^	34.9 (33.4-36.3)	33.0 (29.6-36.4)	0.315	36.7 (34.9-38.6)	32.1 (30.3-34.0)	0.001	0.385	0.158
Monounsaturated fatty acids (g)^2^	29.6 (28.6-30.6)	27.6 (25.2-30.0)	0.144	32.8 (31.4-34.3)	28.4 (27.0-29.9)	<0.001	0.203	0.329
Polyunsaturated fatty acids (g)^2^	16.4 (15.5-17.4)	12.9 (10.6-15.2)	0.006	17.1 (15.8-18.4)	13.2 (12.0-14.5)	<0.001	0.954	0.655
Iodine (μg)^2^	151 (144–159)	140 (123–157)	0.237	159 (149–169)	170 (160–181)	0.113	0.278	0.448
Calcium (mg)^2^	913 (867–960)	966 (855–1078)	0.390	904 (839–967)	919 (854–984)	0.752	0.101	0.072
“with supplement (mg)^2,3^	948 (901–996)	999 (886–1112)	0.418	916 (851–981)	924 (859–990)	0.860	0.023	0.014
Iron (mg)^2^	12.9 (12.5-13.4)	13.4 (12.3-14.5)	0.478	14.3 (13.7-14.8)	14.7 (14.2-15.3)	0.243	0.032	0.032
“with supplement (μg)^2,3^	14.9 (13.9-15.8)	15.3 (12.9-17.6)	0.737	15.9 (14.9-16.9)	15.2 (14.3-16.2)	0.381	0.734	0.996
Vitamin D (μg)^2^	6.6 (6.3-6.9)	6.7 (6.0-7.4)	0.890	6.9 (6.5-7.3)	6.3 (5.9-6.7)	0.024	0.433	0.212
“with supplement (μg)^2,3^	9.9 (9.2-10.5)	10.6 (9.1-12.2)	0.375	8.1 (7.5-8.7)	7.2 (6.6-7.9)	0.071	<0.001	<0.001
Folate (μg)^2^	277 (253–301)	280 (222–338)	0.939	300 (274–326)	327 (300–353)	0.169	0.629	0.946
“with supplement (μg)^2,3^	423 (386–460)	458 (369–547)	0.478	353 (316–389)	370 (333–407)	0.522	<0.000	<0.001
Vitamin B12 (μg)^2^	5.4 (5.1-5.7)	5.6 (4.9-6.4)	0.506	6.0 (5.5-6.4)	5.6 (5.2-6.1)	0.263	0.450	0.675
“with supplement (μg)^2,3^	6.5 (6.1-6.8)	7.0 (6.1-7.8)	0.305	6.4 (5.9-6.8)	5.9 (5.4-6.4)	0.210	0.056	0.015
**Foods**								
Potato/rice/pasta (g)^4^	169 (155–182)	145 (113–176)	0.170	186 (165–207)	211 (190–231)	0.248	0.004	-
Meat/fish (g)^4^	99 (91–107)	110 (92–129)	0.295	125 (113–138)	127 (115–140)	0.992	0.001	-
Vegetables (g)^4^	102 (84–119)	71 (30–112)	0.172	141 (112–169)	149 (121–178)	0.686	0.002	-
**Supplements (%)** ^**5**^								
Multi-vitamin	40.9	51.5	0.258	14.6	13.6	0.841	<0.001	-
Multi-mineral	17.6	15.2	0.731	5.8	2.9	0.498	<0.000	-
Iron	10.8	12.1	0.766	8.7	2.9	0.134	0.058	-

Data are presented as mean/median with 95% CI/min-max, respectively. Further, proportion (%) reporting intake of a supplement the latest two weeks in early pregnant women and a population based referent group.

^1^Mean values are based on reported FFQ frequencies, with adjustment for education (university yes/no), body mass index (3 groups) and smoking (yes/no) in age groups and also for age group in pregnancy status groups using a generalized linear model. Carbohydrate, fat, protein, and alcohol intake are presented as the proportion of total reported energy intake originating from the respective nutrient/alcohol (E%). Differences between groups are tested with Student’s *t*-test.

^2^Energy and nutrient intake increased by 25% to adjust for underreporting resulting from shortening of the FFQ by 25% from the original validated version. Means are adjusted for education (university yes/no), body mass index (3 groups) and smoking (yes/no) and age group as described in footnote 1. Differences between groups are tested with Student’s *t*-test.

^3^Mean values represent estimated adjusted nutrient intake with addition from supplements in those who reported intake of a multivitamin, multimineral, or iron supplement the latest 2 weeks. Addition has been done with the most common content in over-the-counter sold supplements targeting women, *i.e.* calcium 200 mg, iron 18 mg, vitamin D 7.5 μg, folate 200 μg as folic acid, and vitamin B12 2.5 μg. Adjustment and testing are as described in footnote 2.

^4^Mean values are based on reported FFQ frequencies and portion sizes as defined in the basic FFQ validation study [18].

^5^Numbers represent proportion (%) reporting intake of a supplement the latest 2 weeks. Differences in sampling distribution were tested with Pearson’s Chi-square test or Fischer’s exact test if five or fewer observations in a cell.

^6^Residuals from the regression of the nutrient on energy were used for testing (Students *t*-test) according to the residual method described by Willet [27,34].

Mean daily intake (amounts/day) of whole grain, sucrose, total fat, saturated fat, monounsaturated fat, polyunsaturated fat, iodine, calcium, vitamin D, folate, and vitamin B12 from foods did not differ between the pregnant and referent women regardless of standardization by the residual method or not, whereas iron intake from foods tended to be lower in the pregnant women (Table 2).

Nearly half of the pregnant women reported intake of a multivitamin supplement in the preceding 14-day period compared to <15% among referent women (p < 0.001, Table 2). A significantly higher proportion of the pregnant than referent women also reported intake of a multimineral (although not iron) supplement in the previous 14-day period. Addition from supplements led to significantly higher estimated intake of vitamin D and folate and borderline significantly higher intake of calcium and vitamin B12 in the pregnant than referent women, whereas estimated intake of iron supplement evened out the difference in intakes from foods only (Table 2).

**Figure 1:**
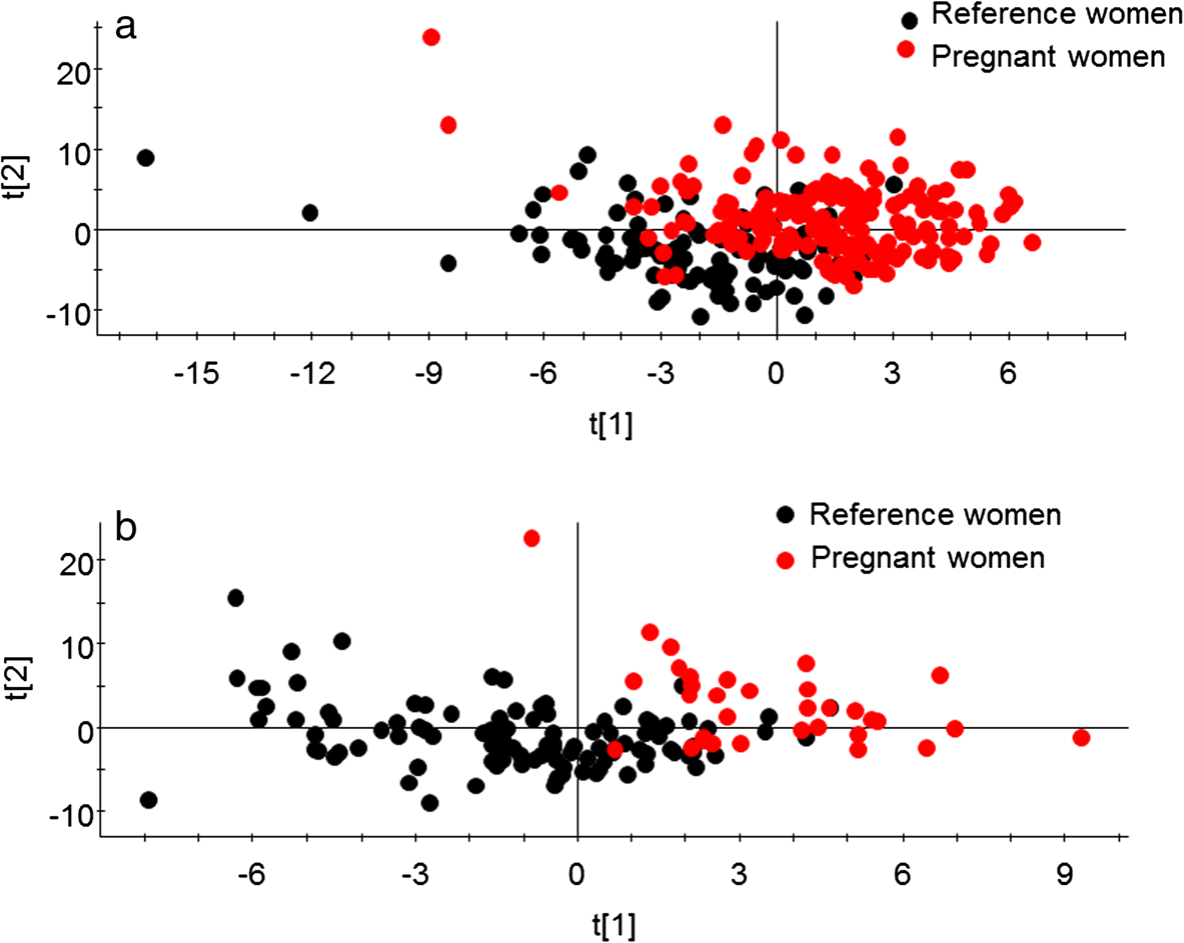
**Clustering of pregnant versus referent women.** Multivariate PLS modelling with the pregnant and referent status as dependent variables and all 66 FFQ foods/food aggregates, nutrients, supplements, and use of tobacco as the block of independent variables. Clustering of participants is displayed in a score loading plot with the two strongest components t[1] and t[2]) on the x- and y-axis for **(a)** 30 year olds and **(b)** 40 year olds.

Multivariate PLS modelling with the pregnant and referent status as dependent variable and all 66 FFQ foods/food aggregates, nutrients, supplements, and tobacco use as the block of independent variables showed a clear tendency of pregnant women clustering separately from the referent women for both the younger and older age group (Figure 1). The variables of importance in the PLS projection confirmed the associations found in the univariate analyses (*i.e*., a higher proportion abstaining from alcohol and more frequent intake of supplements with higher total intake levels of associated nutrients in pregnant women). The referent women were characterized by more frequent and higher alcohol amounts at each drinking occasion, more vegetables, higher coffee intake, higher ratio between monounsaturated and saturated fatty acids, higher intake of beta-carotene, niacin, and cholesterol, and more prevalent use of Swedish snus (snuff).

## Discussion

This study, using multivariate modelling, revealed that women in early pregnancy and a population-based referent group differed with respect to self-reported food and estimated nutrient intake. This difference was a reflection of the dietary pattern in the two groups – early pregnant woman consumed more supplements but less vegetables, potatoes, and meat. In addition, pregnant women had lower use of alcohol, use of tobacco, and intake of iron from food. Both groups reported a low intake from foods of folate and vitamin D compared to the NNR.

For the present study, diet intake was collected with a food frequency questionnaire as this instrument was used for the population study from which the referents were nested. As such, one of the strengths of this study is its similarity of data collection in the pregnant and referent group. However, some limitations need to be addressed. First, the pregnant women were told to answer the FFQ to reflect intake the latest two weeks to avoid monitoring pre-pregnancy dietary habits, whereas the referent women were told to let it reflect their general diet the latest year. One year stability of dietary habits. *i.e.* reliability of the FFQ measured intake, has been found to be very high for participants in the VIP cohort [18], suggesting that comparison of the diet information between the two groups is substantiated in spite of this difference. Secondly, recall bias is a known source of error also in the general VIP cohort [33]. It cannot be excluded that some degree of recall bias exist among the pregnant women and especially so for alcohol, tobacco use and intake of healthy or unhealthy foods. Still the finding of less alcohol and tobacco use accords with other studies and less vegetables and a tendency for more fat does not indicate a severe recall bias among the pregnant women. Thirdly, the study relied on a shortened version of a validated and frequently used food frequency questionnaire, and the major concern relates to limitations associated with recording of diet intake in general and the use of a food frequency questionnaire specifically [27]. Thus, both random and systematic errors may occur due to instrument construction and accuracy of the information given by the respondents. The validity of the original Northern Sweden Diet FFQ has been found to yield similar results to that of other FFQs used in prospective cohort studies [18]. Energy underreporting is prevalent, but to a similar degree as 24-hour recalls, and underreporting is more common among obese participants, low-educated participants, and smokers [28]. To compensate for these factors, mean values were standardized for BMI, education level, and smoking. Using a shortened version of the original FFQ could be seen as a limitation, but the shortened FFQ was unavoidable because, for financial reasons, it had been used in the referent population. To balance the inevitable increment of underreporting, energy and nutrient intakes were adjusted by +25% to facilitate comparisons with what would have been obtained with the longer version, other studies and nutritional recommendations. Furthermore, for group comparisons, energy standardized measures (i.e., the proportion of total energy, E%) were used for energy providing nutrients, and residuals from the regression on energy was used for other nutrients as recommended by Willet [34]. In addition, all groups’ comparisons were standardized for potential confounders, including age, to adjust for differences in food habits by age as well as the unbalanced numbers in the various groups. The latter was unavoidable due to limitations in the basic VIP cohort. Pre-inclusion control confirmed that no pregnant women were among the referent women. It can, however, not be ascertained that none of the referent women were early pregnant, but this is highly unlikely since awareness of pregnancy leads to a routine check-up with a mid-wife in accordance with the Swedish national program instead of a general health screening program.

In an international perspective the mean age in the younger age group of pregnant women (29.2 years), which was the group with most first time pregnant women, may appear high and rise thoughts of an age selection bias. However, this mean age is in accordance with the maternal mean age of 28.4 years in first pregnancy women in Sweden. However, a possible limitation is a potential selection bias of pregnant women as this group included more women with a university education. In part, selection bias in relation to the general population may be the result of the early pregnant women being selected from patients attending antenatal clinics located in the city of Umeå, a university city with a high proportion of residents with a university degree. We do not think that other differences in inclusion conditions between the pregnant and referent women (i.e., the referent women came to the clinic for a general health check up and the pregnant women came to the antenatal clinic) influenced the outcome significantly since participation in both studies was voluntarily and the women likely had similar driving forces influencing their decision to participate. The results from the present study, however, are relevant for the Swedish speaking population only, since women who did not speak or understand Swedish were not included.

Data from the present study, with the precautions described above, agree with data reported in previous studies: diet intake among childbearing-aged women in developed countries is not optimal for pregnancy [35,36]. The European Nutrition and Health Report 2009 [37] found that childbearing-aged women had a low intake of energy, fibre, and micronutrients (e.g., folate, iron, and vitamin D) and a high intake of total and saturated fat. In general, this 2009 report found that the intake of folate and vitamin D from foods was not sufficient to cover the nutritional demands during pregnancy, and supplements were usually required before and during pregnancy [37,38]. These findings are in line with our findings. Similarly, Inskip *et al*. [39] found that only a fraction of women planning a pregnancy reported a nutritional intake reaching the recommendations for women before and during pregnancy, leading them to claim that greater efforts are needed to publicize pre-pregnancy recommendations [39]. Clearly, the evidence from the European Nutrition and Health Report, Inskip *et al*., and our study suggests that effective dietary counselling during antenatal care is an important task for healthcare providers.

As in previous studies from Sweden in women in childbearing age [7], this study found that early pregnant women had a low intake of vitamin D and folate from foods. The average intake of folate among pregnant women was estimated to <300 μg/day, an amount that is close to the mean intake of 247 μg/day reported for 31–44 year old women in Sweden [7]. Since folate is needed for DNA and RNA synthesis in the foetus, a major determinate of the outcome of pregnancy, low levels of folate places the foetus at risk for congenital malformations and the mother and new born at risk due to complications during pregnancy [11]. When considering genetic variations – *i.e.*, in the methylenetetrahydrofolate reductase (MTHFR) gene – that impair folate function and status [21], our findings support a need for supplementation even in populations with access to good quality food. According to NNR, women of childbearing age should supplement their nutrition with 400 μg of folic acid per day and this should be increased to 500 μg per day during pregnancy. In addition, the NNR recommends that women of childbearing age should consume 10 μg of vitamin D per day and for people with little or no sun exposure per day vitamin D intake should be increased to 20 μg per day. In our study, these recommended levels were not reached without supplementation. Our participants, however, seemed to satisfy the NNR’s recommendation of 900 mg of calcium from food per day [3], an amount that ensures an adequate supply for proper foetal skeletal development. Inadequate intake of either calcium or vitamin D can lead to disturbances in mineralized tissues of the foetus [13]. In our study, the pregnant group reported inadequate dietary intake of iron, less than 15 mg of iron per day (NNR recommendation), although this level was reached when supplements were added. Iron balance requires iron stores of approximately 500 mg at the start of pregnancy and the composition of meals influences the use of the dietary iron [3].

Intake of multivitamin supplements during the previous two weeks in the pregnant women was significantly higher than in the referent women. However, it was surprising that only half of the pregnant women reported to have taken a vitamin supplement the previous two weeks in spite of information given from authorities and in the antenatal care. This may reflect underreporting by women taking supplements with a single nutrient, such as folate, since the question in the questionnaire was phrased as a multivitamin, or that it is difficult to achieve compliance with the recommended increased intake of folate even though the risks associated with low levels of folate are dramatic and well documented. Combined with Hure *et al*.’s [40] finding, our observation that women do not appear to consume a wide variety of nutritious foods when planning to become pregnant or during pregnancy calls for targeted dietary counselling during antenatal clinic visits.

Somewhat surprisingly, the women in the pregnant group did not report a higher intake of total energy than the referents, a finding that means that these women were not adhering to the recommendations for additional energy intake during early pregnancy [3]. We can only speculate why these women reported a slightly lower energy intake as we anticipated that pregnant women were motivated to report their dietary intake carefully. The most likely explanation for this finding is that the women had not yet increased their energy intake and may have been too nauseated during this early stage of pregnancy to increase their energy intake [2]. The finding of a lower energy intake suggests that it may be difficult to maintain sufficient energy intake when suffering from morning sickness, but it is beyond the scope of this paper to address the issue of counselling on sufficient energy intake during the early phase of pregnancy and not only prevention of excessive gestational weight gain.

## Conclusions

Women in early pregnancy differed in their dietary intake compared to non-pregnant women as they had a lower intake of vegetables, potato/rice/pasta, meat/fish, and alcohol and had a higher and more frequent intake of supplements. The low intake of folate, vitamin D, and iron from food are of special concern. Though the cross-sectional study design does not allow for a distinction if the pregnant women have changed their dietary habits as a result of pregnancy or if they had a different diet *per se*, generalization to pregnant women visiting antenatal care is justified in a Swedish context. As midwives in antenatal care have the opportunity to influence women to practice good lifestyle choices during early pregnancy, their counselling should be informed by the latest research. However, additional studies are needed to address the status of nutrition intake from a broad multicultural perspective and in a longitudinal perspective.

## Abbreviations

BMI: Body mass index
CI: Confidence interval
DNA: Deoxyribonucleic acid
FFQ: Food frequency questionnaire
NNR: The Nordic Nutritional Recommendations
NSDD: Northern Sweden Diet Database
PLS: Partial Least Square regression
PregNut: Pregnancy and Nutritional Cohort
RNA: Ribonucleic acid
VIP: Västerbotten Intervention Programme
